# Wills of Medical Men

**Published:** 1921-03-26

**Authors:** 


### Wills of Medical Men.

Mr. T. W. Iddon, M.B., of Southport, Lancs, left £28,286.

Mr. George Herbert Spencer, M.R.C.S., L.R.C.P., of Farnworth, Bolton, surgeon to several collieries, left £7,116.

Dr. Willoughby Turner, O.B.E., M.D., F.R.C.S., of Hove, left £2,000 to the Sussex County Hospital; his estate is valued at £16,585.

Surgeon-General W. G. Don, of Canfield Gardens, Hampstead, President of the Caledonian Society of London, has left personal estate valued at £3,963.

Sir Frederick Taylor, Bart., M.D., at one time Examiner in Medicine in the Universities of Durham, London, Birmingham, Cambridge, and Belfast, and ex-President of the Royal College of Physicians, left estate valued at £20,984.

Dr. James King Kerr, J.P., of Glenallans, Knock, Belfast, a former Chairman of the Belfast Public Health Committee, has left property valued at £12,893.

Mr. Alexander Cosgrave, L.R.C.P., M.R.C.S., of Bolton, Lancashire, formerly Surgeon-Major 3rd East Lancs Brigade, R.F.A., T.F., left estate to the value of £5,478.

Dr. Robert Munro, M.D., LL.D., F.R.S.E., of Elmbank, Largs, N.B., Secretary of the Society of Antiquaries of Scotland, aged eighty-four, left personal property valued at £38,959.

Mr. Samuel Stretton, M.R.C.S., L.S.A., of Droitwich, Senior Consulting Surgeon to the Kidderminster Infirmary, who saw service in military hospitals during the Crimean War, left estate to the gross value of £8,868.

